# Discrete-event simulation modeling for housing of homeless populations

**DOI:** 10.1371/journal.pone.0284336

**Published:** 2023-04-26

**Authors:** Dashi I. Singham, Jennifer Lucky, Stephanie Reinauer

**Affiliations:** 1 Operations Research Department, Naval Postgraduate School, Monterey, CA, United States of America; 2 Alameda County, Office of Homeless Care and Coordination, Oakland, CA, United States of America; 3 Abt Associates, Bethesda, MD, United States of America; Universitat Politecnica de Catalunya, SPAIN

## Abstract

The San Francisco Bay Area has experienced a rapid rise in homelessness over the past decade. There is a critical need for quantitative analysis to help determine how to increase the amount of housing to meet the needs of people experiencing homelessness. Noting that the shortage of housing available through the homelessness response system can be modeled as a queue, we propose a discrete-event simulation to model the long-term flow of people through the homelessness response system. The model takes as input the rate of additional housing and shelter available each year and delivers as output the predicted number of people housed, sheltered, or unsheltered in the system. We worked with a team of stakeholders to analyze the data and processes for Alameda County in California and use this information to build and calibrate two simulation models. One model looks at aggregate need for housing, while the other differentiates the housing needs of the population into eight different types. The model suggests that a large investment in permanent housing and an initial ramp up of shelter is needed to solve unsheltered homelessness and accommodate future inflow to the system.

## 1 Introduction

Many parts of the US have faced housing crises, where rising home and rent prices have led to more people unable to afford housing. The San Francisco Bay Area has been notably affected. Alameda County is located east of San Francisco and includes the cities of Oakland and Berkeley, while having a population of approximately 1.7 million. This county has faced rapidly increasing numbers of persons experiencing homelessness in the past decade with recent estimates of approximately 13,000 households needing help each year. The homelessness response system is defined as the set of housing, shelter, and services dedicated to people experiencing homelessness [1]. The county has devoted significant resources to increase housing and shelter for people experiencing homelessness. Housing refers to a housing unit or a subsidy that allows someone to have a place to live without time limits, though this housing may be supported by resources in the homelessness response system. Shelter refers to temporary accommodations that provide a safe, temporary place to stay until a permanent housing solution becomes available. There exists a large number of unsheltered people waiting for both housing and shelter due to a lack of space in the system. In the Bay Area, it is this recent increase in unsheltered homelessness that has led to even higher levels of concern. The commitment from the community to solving this crisis has led to a number of new proposed solutions, and quantitative methods are critical to evaluating the potential effectiveness of these solutions.

Our goal in this work is to introduce discrete-event simulation as a method for modeling the flow of people through the homeless system and predicting the effects of investing in increased housing on the unsheltered population. Discrete-event simulation is a key tool for modeling the flow of entities through a system with constrained resources. Discrete-event simulation is also easily able to incorporate randomness in the arrival rate of people to the system, or uncertainty in the length of time they will occupy a particular resource. To the best of our knowledge, this study is the first approach that uses discrete-event simulation to model the flow of people through an entire homelessness response system as a queueing system.

We identify some of the key aspects of this queueing problem. The primary resource is housing, which is in limited supply due to space constraints and high financial costs for building new units or providing rental subsidies for existing housing. Additionally, turnover in housing units operated by the homelessness response system is low because these units are designed to provide a permanent place for formerly homeless people to reside. The lack of resources to create additional housing opportunities (units or subsidies) at a rate fast enough to keep up with new additions to the homeless population is one reason for the current bottlenecks in the system. A secondary resource is emergency shelter, which is designed to provide a temporary place to stay until a person’s homelessness can be resolved. While the intent is for people to stay in shelter for a matter of weeks or at most a few months while permanent housing is being arranged, in reality people may stay far longer due to the lack of access to housing downstream. The shortage of housing and shelter has led to a large number of unsheltered people who face limited options, and we model this group as a queue for housing and shelter resources. Unsheltered homelessness is defined as people residing in a place not designed for regular sleeping accommodations. Our model focuses on people and housing contained within Alameda County’s homeless response system, so we do not directly consider homeless people who are not seeking homeless assistance resources or the effects of housing limitations in the general real estate market.

This approach to studying the system is clearly a queueing problem: there are arrivals to a system with constrained resources, and the large queue for these resources is the source of this crisis. The homelessness response system can be classified as unstable from a queueing standpoint because the rate of arrivals to the homeless response system is higher than the rate people can be served and placed in housing. This queueing system also incorporates complex aspects such as blocking, where people cannot leave one resource because there is no capacity for them at the next resource. Blocking and instability make analytical formulations of queueing models intractable in most cases, so discrete-event simulation is a natural option for modeling this complex system.

The objective of this research is to build a simulation model to test different investment policy scenarios subject to uncertainty in the system. Policymakers must decide how much additional housing and shelter can be generated and in what time frame, given limited resources and pressure to alleviate the suffering of people experiencing unsheltered homelessness. It is clear that the inventory of emergency shelter must be increased in the short term to reduce the growing unsheltered homeless population, but in the long run the goal is to have most resources invested in housing and have only minimal necessary shelter available as a safety net when suitable housing opportunities aren’t immediately available. One idea is to start with a surge increase of emergency shelter to rapidly reduce the unsheltered homeless population, and when levels of housing inventory catch up to current need, convert excess shelter to permanent housing.

There are many desired objectives, but one goal is referred to as “functional zero”. Functional zero does not mean that no one becomes homeless, but that the homelessness response system has sufficient resources to quickly rehouse people when they do experience homelessness, i.e., there is no unmet need. Unmet need is defined as the number in the queue plus the number in emergency shelter awaiting housing for each pathway. The goal of functional zero is in contrast with the current state of affairs, where it can take years from the time a homeless person seeks housing to the time permanent housing is obtained. Thus, the goal is not only to bring the number of unsheltered down to zero, but to maintain sufficient housing inventory so that less emergency shelter is needed.

An additional challenge is communicating proposed investment options to decision makers. Given the costs associated with solving the homeless crisis, accurate models are needed to justify high levels of investment in homeless-dedicated housing. Policymakers may have other priorities for use of limited funds, or may balk at the large cost of generating housing opportunities. Constituents may prefer to shelter people as quickly as possible to reduce the number of unsheltered people living on the streets. However, undesirable shelter conditions may deter people from entering shelter, especially if there is no clear or timely pathway to housing. An accurate simulation model could show the long-term effects of investment in housing on the overall homeless population to aid in decision making.

Some of our contributions are generalizable beyond the geographical region considered in this paper. First, the idea of using queueing simulations to model unsheltered homeless populations on a large scale is new (to the best of our knowledge). Thus, we hope this work inspires similar quantitative models of humanitarian crises. At the very minimum, other communities with housing shortages can build similar queueing models to determine the effects of investment policies on the number of homeless people. In areas facing similar types of housing resources and individual pathways through the system, details from our model can be directly applied using different data inputs, but in many localities the pathways will have to be modified to meet the requirements of that region.

This paper presents two simulation models developed in conjunction with the Alameda County Office of Homeless Care and Coordination. Section 2 outlines related literature, and Section 3 details the data collection efforts used to calibrate the simulation models. Section 4 describes the first simulation model which aggregates all types of housing into a single category, while Section 5 presents a model that includes eight different pathways through the system. Results of the simulation models are presented in both Section 4 and Section 5. Section 6 contains concluding thoughts and future work.

## 2 Literature review

The combination of simulation and optimization has been widely used to address the challenges associated with complex societal problems requiring local government coordination (i.e, planning emergency response infrastructure [2]). Simulation can often be used to test the effects of potential interventions in healthcare and human systems, for example, the work in [3] studies medical clinics and [4] models an adoption matching process. Additionally, the problems for constructing and allocating affordable housing has been studied under community based operations research [5].

There has been much work to develop analytical and statistical models for various aspects of the homeless population. The work in [6] used an agent-based model for tuberculosis outbreaks in shelters, while [7] develop spatial models for tuberculosis outbreaks among the San Francisco homeless population. In [8], a regression model is developed to identify trajectories of increased functional impairment among people experiencing homelessness in Oakland, CA. Of particular interest in Oakland is research into the reasons why people of color experience homelessness at disproportional rates. [9] surveyed people in Oakland to assess levels of overt racism and structural racism in the process of obtaining housing.

The model in [10] projects the number of rooms needed to isolate and house members of the homeless population during a COVID-19 surge in Austin, TX. Developing good estimates of the needs of the homeless population during changing conditions is crucial to resource planning, and it was important to take into account the changes in arrivals to the system during the pandemic. The authors in [11] develop a discrete-event simulation in Simio to model the process of COVID-19 testing and determine where potential improvements could be made to increase testing capacity. The effect of COVID-19 outbreaks in homeless shelters was also studied in [12], which suggests a need for non-congregate shelter as opposed to high density congregate shelter where measures to prevent an outbreak may be unsuccessful.

Models for matching homeless individuals with housing have been studied in great detail. For example, [13] consider the quality of a match between an individual and housing provider, and solve an optimization problem to find the best matching using a number of heuristic methods. The decision on the number and type of options offered in social service settings was analyzed in [14], whereby it is sometimes optimal to offer a less diverse set of services and higher advisory levels to ensure people get matched with the correct service levels. In [15], the authors developed an extensive queueing model to propose interpretable policies that attempt to ensure fairness across groups in allocating limited housing resources.

Discrete-event simulation was used in [16] to model the flow of homeless persons through a health clinic in Lexington, KY. Various staffing levels and random processing distributions were used to estimate the effect on waiting times in the queue. Simulation was also used in [17] to assess the quality of a partially observable Markov decision process to optimize the choice of sequential interventions to help homeless youths using social networks. Like some of the research mentioned above, our work uses discrete-event simulation, but models the overall flow of people through the entire homelessness response system over a long-term period of years, rather than focusing on a particular clinic or shelter in the short term.

## 3 Model calibration

To the best of our knowledge, discrete-event simulation has not been used to model the flow of people through a homelessness response system including the final stages of permanent housing. Queueing models provide a natural framework for analyzing the shortage of housing in the homeless response system which is causing a large number of people to be unsheltered. Given the relative complexities and limitations of moving people through the system, discrete-event simulation is an ideal tool to model this complex process. We build two discrete-event simulation models using Simio simulation software to incorporate detailed data about the system.

The homelessness response system describes the process from the time that a person is identified as needing assistance, to the time they exit the system to permanent housing. While there are many administrative steps involved in entering the system and receiving services, we focus our model on the major stages of emergency shelter and housing, which are the primary bottlenecks in the system. In Alameda County, the homelessness response system is unstable from a queueing perspective. Thus, standard queueing approximations will not necessarily hold, and simulation will allow for the flexibility to model the resulting crisis due to this instability. Simulation also allows for easy scenario analysis to model different configurations of investment policies to test the effects of varying rates of adding new shelter and housing inventory over time.

The authors conducted a study of the data in the system in conjunction with Abt Associates (a HUD technical assistance provider) members of Alameda County’s Office of Homeless Care and Coordination, as well as other local nonprofit leaders and stakeholders. Data was pulled from HMIS (Homeless Management Information System) to obtain estimates of the number of households and individuals served by the system annually as well as rates of new homelessness, housing and shelter inventory levels, and rates of returns to the system. This data was used in a systems modeling study to estimate the yearly inflow/outflow through the system. This systems model was constructed by Abt Associates, and we use this model to calibrate model logic in the simulation study.

The point-in-time (PIT) count is an estimate of the number of homeless people within a given community conducted over a single night. The PIT count is hard to obtain and can be collected using grid searches of cities. Estimates of the homeless population have also involved telephone surveys [18]. PIT counts are critical to calibrating our model to estimate the number of people in the system at the start of the simulation. The authors in [19] develop an alternative method for estimating the number of homeless persons by looking at data from deceased persons. This method estimates the hidden homeless population, consisting of people who may exist in settings not observed or counted using traditional methods because they are not in direct contact with care systems. In 2021 the Oakland-Berkeley-Alameda County Continuum of Care (CoC) analyzed data in Alameda County to determine the extent to which racial disparities are present in the homeless system, and the data analysis and findings from this report helped to inform the parameters of our model [20].

Of those that leave the homelessness response system, approximately 17% will return to the system in two years, further increasing the inflow. In Alameda County, the current arrival rate of new people to the system, as estimated from HMIS data, is approximately 3,500/year, or around 10/day. This is the rate used as the base arrival rate. Given the independence in individual arrivals to the system, we use a Poisson process to generate arrivals (the time between arrivals has an exponential distribution with mean 1/10 days). In the greater Bay Area region, this rate has been driven by rapidly increasing housing prices. The systems modeling study assumed an increase in new arrivals to the system in the first two years (20% increase in 2022 followed by a 10% increase in 2023). This is followed by a stabilization with no change to the arrival rate in 2024, and a decline in arrivals in the last years (a 10% decrease in both 2025 and 2026) due to proposed prevention methods. Funding for homelessness prevention is separate from the funding for shelter and housing, and refers to services and financial assistance provided to people before they lose the place where they are living and become homeless. Systems modeling predicts the need for an estimated $18 million for homelessness prevention programs operated by the homelessness response system. In the simulation model, we will use a non-homogeneous Poisson process to model the changing arrival rate over time. Additionally, while there was much effort to analyze data relating to households with children, we focus this model on adult-only households since those comprise the vast majority (approximately 90%) of homeless households in Alameda County.

The COVID-19 pandemic was anticipated to lead to a surge in homelessness in part because of job losses and economic conditions, but also because shelters could no longer operate at full capacity. However, the scheduled PIT Count in 2021 was disrupted during the COVID-19 pandemic, making it hard to update the estimated number of unsheltered people. On a positive note, in California, the pandemic resulted in Project Roomkey, where vacant hotels and facilities were used to house and isolate homeless persons at risk for COVID-19. [21] analyzes the effect of Project Roomkey and highlights that it was successful in sheltering thousands of people and moving them into permanent housing at much higher rates than standard congregate shelters.

We use the available data to calibrate two simulation models. The first simulation model presented in Section 4 studies the aggregate flow of the homeless population through Alameda County without distinguishing between different types of housing needs. The second simulation model in Section 5 incorporates different pathways (combinations of shelter and types of housing resources used) that could result in people exiting homelessness to housing based on varying household needs. All simulation results and code used to generate the plots in this paper are available on a public repository.

## 4 Aggregate model

**Fig 1:**
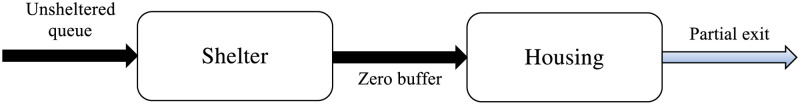
Aggregate housing and shelter system. The blue arrow “Partial exit” means most people will stay permanently housed in county resources and not exit the homeless response system. But some percentage of people may leave the system which frees up their housing unit.

We first build a model of the aggregate system which considers all types of housing as a single type of resource. This simpler model will enable us to assess in general terms how much housing is needed to eliminate unsheltered homelessness over the next five years. Fig 1 shows the simplest possible layout. People arrive to the system seeking housing. If housing isn’t available, they attempt to stay in shelter. If shelter is not available, then they wait in the queue for shelter. This queue represents the current unsheltered population. Between shelter and housing we do not model a queue, because people wait for housing to become available before leaving shelter. Thus, the shelter server is often “blocked” because it cannot release people if there is no housing available for them. In reality, people may leave shelter for various reasons and return to the homeless population, but their spot would immediately be filled anyway by someone else given the current high levels of homelessness.

The shelter resource is not a typical server because rather than each person having a specified time in the server, people wait until a housing resource becomes available before leaving. Similarly, the housing server is not a typical server because many people stay in the system in housing supported by the homelessness response system indefinitely, though they are no longer homeless. A successful housing placement often results in a permanent housing accommodation. As mentioned in Section 3, a percentage of people return to homelessness after gaining and then losing permanent housing. We model the time spent in the housing server as a random triangular distribution with minimum 0, mode 6 years, and maximum 8 years to model long stays in this server (note the simulation is run for a total of 5 years). This choice was based on discussions with system experts who stated that most people remain in housing supported by the homelessness response system, but approximately 17% of the people who move into permanent housing return to homelessness within 2 years. Triangular distributions are useful for modeling finite (bounded) service processes with unimodal distributions. Our distribution choice models approximately 52% of people leaving in 5 years which approximates this rate of leaving, but this distribution could be adjusted for models attempting to predict for time horizons longer than 5 years. The high possibility of an indefinite stay in housing makes it even more difficult to obtain stability (in the queueing sense) because the outflow from the housing server is much lower than the inflow to the system. Stability is achieved when the inflow is less than the outflow to the system.

Thus, one potential solution is to continually increase the amount of housing inventory to accommodate increased demand and limited outflow. One objective is to increase overall shelter and housing units to decrease the unsheltered population. In the ideal long-term case, people would primarily be housed in permanent housing, and minimal shelter would exist to handle the incidental short-term backlog as people wait for housing. However, given the currently high number of unsheltered homeless people in Alameda County, it may make sense to have a surge of shelter created to temporarily give people a place to stay while long-term housing opportunities are being developed. It may be possible to then convert some of the shelter to permanent housing as the unsheltered population decreases.

We can see the effects of various investment policies on the system. These types of policies may not always be feasible and are subject to budgetary constraints, but the simulation model can be used to determine the corresponding effects on the population experiencing homelessness. Under the guidance of Abt Associates, representatives from Alameda County’s Office of Homeless Care and Coordination and other regional partners used systems modeling to identify the shelter and housing inventory that would be needed for the countywide homelessness response system to reach functional zero in five years. Table 1 shows rounded values of the proposed plan to build shelter and housing over a five-year period. Shelter would increase in initial years, but then decrease over time as it is transitioned to permanent housing, while housing inventory increases steadily.

**Table 1:** Approximate investment plan derived from the stakeholder group. Units are total numbers of adults accommodated at the beginning of the year.

Year	Total Shelter	Total Housing
2022	1,500	4,000
2023	2,500	6,000
2024	3,200	9,600
2025	3,000	13,600
2026	1,600	19,300
2027	1,200	24,000

**Fig 2:**
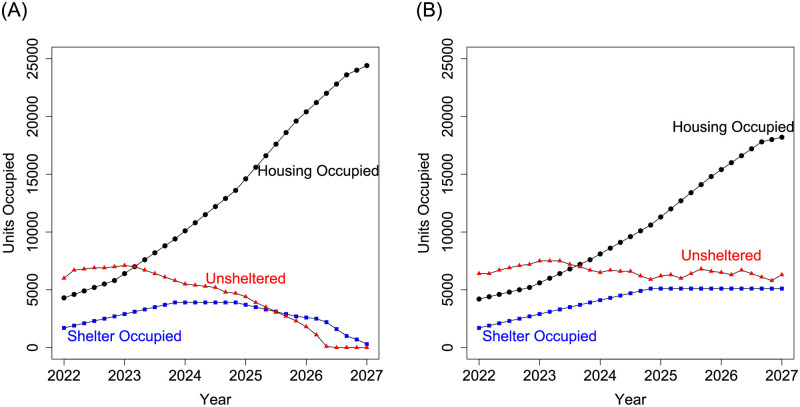
Left: Aggregate simulation results using the investment plan in Table 1. Right: modified plan with 70% investment in housing compared to Table 1, and no decline in shelter. Both plots are the result of a single replication of the simulation model.

Fig 2 shows the results of two possible investment policies using the aggregate simulation model. These results come from a single replication of each simulation, so are representative of a possible reasonable trajectory, but not the estimated average trajectory. The left plot shows a scenario using the proposed plan in Table 1. With heavy investment in housing, we see a steep increase in occupied housing over the five-year period. The amount of shelter available ramps up initially, then declines in later years. Because of the heavy investment in housing, the unsheltered population decreases over time and eventually the goal of functional zero is met.

The right plot of Fig 2 shows an alternate simulation where there is only 70% investment in housing compared to the values in Table 1. There is some additional ramp up of shelter, and no decline or conversion from shelter in later years. We see that such a plan stabilizes the number of unsheltered people, but is not able to bring it down to zero. Thus, without rapid increases in housing and shelter to deal with current and future unmet need, functional zero is unlikely to be achieved within five years.

**Fig 3:**
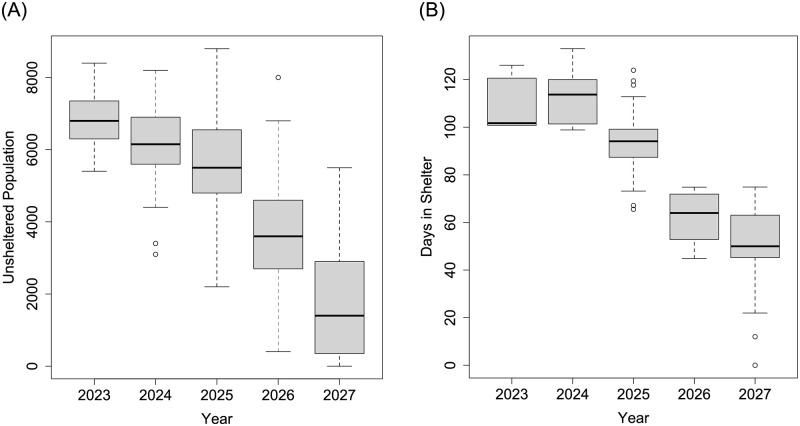
Left: Boxplots showing the unsheltered population over time using 100 replications of the aggregated model. Right: Boxplots showing the number of days in spent in shelter over time using 100 replications of the aggregate model. Both plots use the investment plan according to Table 1.

Because the system is so overloaded, the amount of housing and shelter occupied is generally the same across model replications (the resources are always filled to maximum capacity). However, some aspects of the model display variability upon running multiple replications. Fig 3 shows the results of running 100 replications of the aggregate model according to the investment plan in Table 1. The left plot displays parallel boxplots for the number of unsheltered over the years, whereby we see some replications getting close to bringing the unsheltered down to zero, but there is still high variability. The right plot of Fig 3 displays boxplots for the number of days spent in shelter waiting for housing, and this also decreases over time as more housing is built.

The aggregate model can be used to get a sense of the overall volume of new housing needed to meet the long-term goals of Alameda County’s homelessness response system, and we see that approximately 24,000 units of housing are needed over 5 years if the current rate of inflow to the system remains the same. Next, we present the detailed model which differentiates individual pathways through the homelessness response system and models separate queues for each type of housing. This analysis can inform the allocation of resources towards certain types of housing options.

## 5 Detailed pathway model

**Fig 4:**
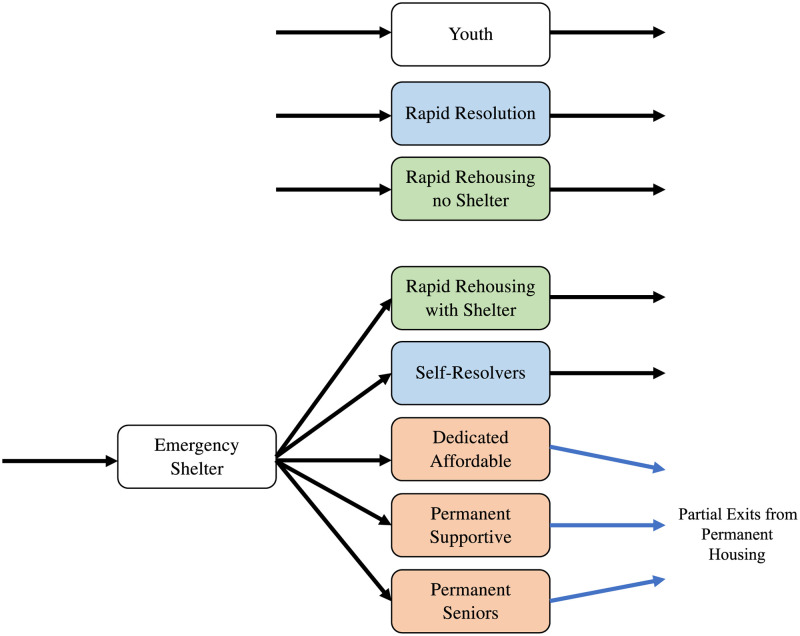
Detailed model of pathways through the system.

While the aggregate model gives some indication of total rates needed for generating housing and shelter inventory, it does not take into account the nuanced pathways taken by different types of people through the system. People arriving to the system have very different needs. Some may require permanent housing with medical and social service assistance, while others, such as those on a fixed income, may simply need additional funds to pay monthly rent. Fig 4 shows a simplified layout of the pathways through the homelessness response system in Alameda County. There are some pathways that do not involve stays in emergency shelter in which people go directly from unsheltered homelessness to a housing resource. Transitional Housing for youth is designed to temporarily house young adults ages 18 to 24 experiencing homelessness, and provides supportive services such as case management, health care and employment. Rapid resolution (RR-Short) offers resources to those who are in the process of becoming homeless, but may be able to avoid entry to the homeless system by receiving help finding housing resources, funds, and transportation. Rapid resolution and self-resolvers (SR) (the blue servers) involve minimal intervention from the system, but self-resolvers either will be able to resolve their homelessness quickly through their personal networks, or will leave the system completely.

Rapid rehousing with shelter (RRH) (green servers) are time-limited subsidies designed for people who are in need of short term housing assistance who are likely to increase their income within a defined time frame. Some people in this category require a stay in emergency shelter, while Rapid rehousing with no shelter (RR-Long) provides assistance to employed people who are struggling to afford their rent, and are unlikely to increase their income due to health, disability, or education reasons.

Finally, the orange servers are those requiring the most resources as they are the highest cost and house people for long periods of time. These pathways are for those who require permanent or long-term housing solutions. Dedicated affordable (DA) housing provides housing to low-income residents who are at risk of homelessness. Permanent supportive housing (PSH) includes permanent subsidies linked to income and are aligned with services to keep residents in stable housing, while permanent supportive housing for older/frail adults (PSH-Seniors) is allocated a separate pathway and provides a higher level of services for people with cognitive or physical disabilities. The lack of housing in this orange category is a major part of the current crisis because this type of investment is costly and time consuming and currently has high demand. Additionally, there is low turnover in this category. Most people may not exit the housing resource ever (thus there are only “partial exits” from these servers).

The detailed model has many enhancements from the aggregate model to account for the differences in pathways. The different types of arrivals each have their own arrival rate proportional to their representation in the system. Each type of resource has a unique distribution for the amount of time a person occupies the housing. For example, permanent supportive housing and dedicated affordable housing are generally expected to be occupied by the same person for years, while rapid resolution usually provides financial support on the order of months. Details on each type of pathway are included in Table 2.

**Table 2:** Pathway population details including proportion of population, *average* time in shelter, and distribution of time in housing where ‘Tri’ refers to a triangular distribution with given minimum, mode, and maximum and ‘Unif’ refers to the uniform distribution with given minimum and maximum.

Pathway	Prop. of pop.	Shelter Time	Housing Time
Youth Transitional (Youth)	2%	N/A	Unif(1,2) years
Rapid Resolution—Long term (RR-Long)	10%	N/A	Tri(2,6,8) years
Rapid Resolution—Short term (RR-Short)	10%	N/A	3 mos
Rapid Rehousing (RRH)	15%	Until hsng avail	Unif(1,2) years
Self Resolution (SR)	10%	5 mos	N/A
Dedicated Affordable (DA)	28%	Until hsng avail	Tri(2,6,8) years
Permanent Supportive Hsng (PSH)	15%	Until hsng avail	Tri(2,6,8) years
Permanent Supportive Hsng, Seniors (PSH-Seniors)	10%	Until hsng avail	Tri(2,6,8) years

Additionally, each type of arrival to the system is assigned a priority. Emergency shelter space is often limited, so priority is given to those with the most physical need like people requesting resources from PSH and PSH-Seniors. Each type of arrival also has its own need for shelter. Some people do not need any shelter, some need it for a limited amount of time, while others may need to stay in shelter indefinitely until an exit to housing becomes available. As in the aggregate model, the shelter server may often be blocked if no appropriate housing is available. People who may require permanent supportive housing will wait in shelter indefinitely until housing is available, and this may prevent those who only need temporary shelter for a few months (or even weeks) from obtaining shelter.

In the detailed model, we take into account the number of resources available for each pathway over time. The proposed investment policies will increase the number of resources available each year attempting to match the proportions of relative need in the system. The overall intention is to reduce the number of unsheltered people, and eventually decreasing even the amount of emergency shelter needed. The emergency shelter serves as a backstop for those who are unable to access housing, but given current capacity limits on shelter space there still are thousands of unsheltered people in the county. Similar to the aggregate model, as housing investment increases, we can create flow through shelter through increasing exits to housing.

Table 3 contains one such investment policy proposed by the systems model results. We call this policy IP100 and this will form the baseline for generating alternate policies. It attempts to increase resources proportionally to the approximate population of people requiring the pathway. As in the aggregate model, the total shelter will increase initially to decrease the number of unsheltered people, but will decrease in later years as more housing becomes available.

**Table 3:** Approximate Investment Policy (IP100) suggested by the stakeholder team to match proportional need. Values are total units to exist by the end of the year (not incremental new units).

Year	Shelter	Youth	RR-Long	RR-Short	RRH	DA	PSH	PSH-S
2023	2,652	104	677	130	1,120	1,459	3,351	521
2024	3.221	121	1459	152	1,305	3,085	4,054	1,086
2025	2,984	138	2,260	173	1,488	4,869	4,837	1,691
2026	1,652	195	3,416	244	2,100	7,359	6,013	2,532
2027	1,253	173	4,368	216	1,857	9,411	6,914	3,194

**Fig 5:**
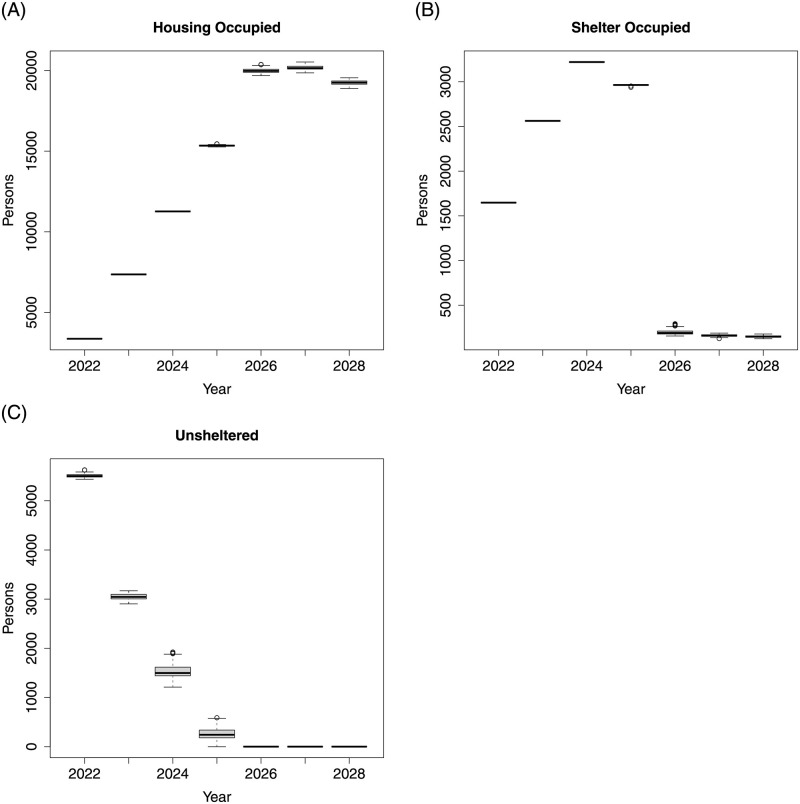
Boxplots showing uncertainty in simulation trajectories for housing occupied, shelter occupied, and the unsheltered population for the investment policy IP100 in Table 3.

We run 100 replications of the detailed model using the investment policy in Table 3 to see the effect of parameter uncertainty on the overall results of the investment policy. Fig 5 shows parallel boxplots for each year representing the uncertainty in the predicted values of the amount of housing occupied, the amount of shelter occupied, and the number of unsheltered people. Because the system is resource constrained, there is not much uncertainty relative to the overall number of people in the system. Housing will generally be occupied at full capacity.

At the end of the five-year period in Fig 5 we see a possible drop in housing occupied due to exits from the homeless response system. Shelter occupied is also at its limit for the first few years, though once housing increases, it drops drastically and mainly serves as a backstop when housing is unavailable for a particular pathway. We see some uncertainty in the number of unsheltered people, as this is the combined number in the queue for all the pathways. We expect there to be some variability in the number of unsheltered people prior to the system reaching functional zero. Just as in the aggregate model, the amount of housing and shelter occupied are generally at their maximum values so there is not much uncertainty. Compared to the aggregate model, the uncertainty in the number of unsheltered is lower because the detailed model does a better job getting people into housing, in part because some of the housing pathways have shorter time spent with the resource than the aggregate model assumed. The aggregate model was more conservative in assuming worst-case resource usage for all people, rather than being differentiated by varying need as in the detailed model.

**Fig 6:**
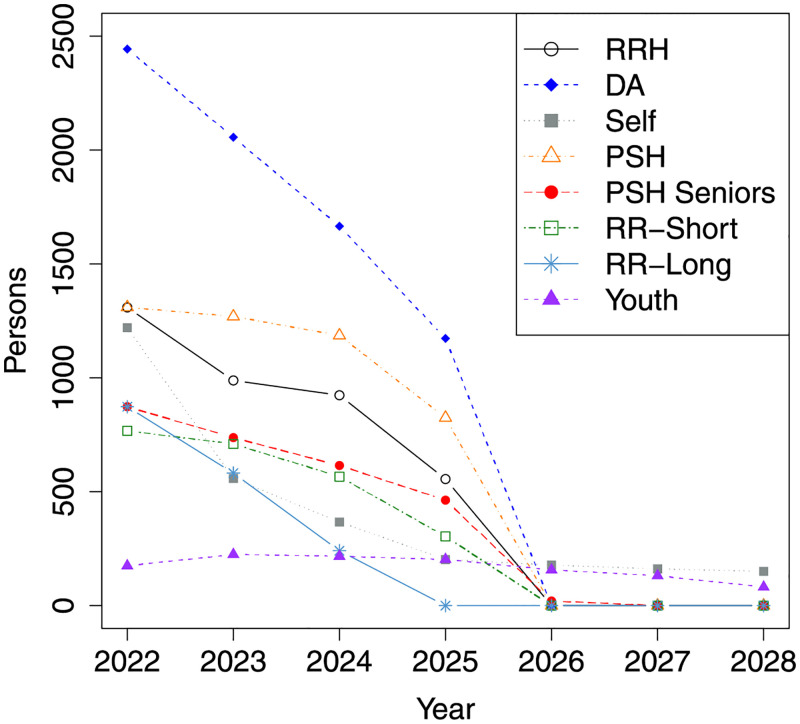
Unmet need for each pathway, each point averaged over 100 independent stochastic replications, using the investment policy in Table 3 (IP100).

The proposed investment plan in Table 3 costs an estimated $2.5 billion, which covers operational costs, not including development (or capital costs). The detailed model allows us to differentiate between types of housing allocation policies and see the effect of prioritizing different types of need. Fig 6 shows the amount of unmet need for each pathway over time by averaging over 100 independent replications of the detailed model using the investment policy IP100. Essentially this is the number of people who have not reached the final stage of housing whereby they would be marked as a successful completion.

We see the unmet need decreases over time and for the most part goes to zero after four years. Both youth and self-resolvers will continue to have unmet need in the system as they arrive and may find constraints on housing and emergency shelter as the system reaches a steady state. The investment policy in Table 3 is based on allocating proportionally to population needs, so fewer resources are initially allocated to youth transitional housing. Self-resolvers don’t require housing investment, just shelter, so there will always be some self-resolvers in shelter while they arrange their resolution.

**Fig 7:**
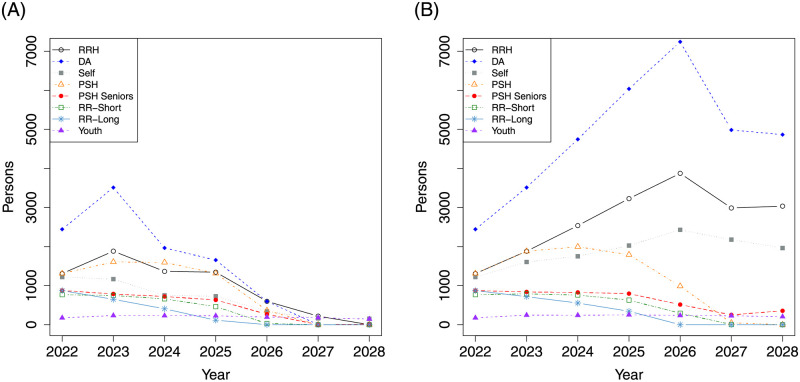
Left: Unmet need using investment policy IP90 when investment is 90% of IP100. Right: Unmet need using investment policy IP80 when investment is 80% of IP100.

To see how sensitive these trajectories are to the investment policy in Table 3, we test what would occur if investment happened at the slower rates of 90% and 80% of the total units built in IP100 (call these policies IP90 and IP80). These results are plotted in Fig 7. By scaling down the number of units available, we see that it takes longer to reach functional zero and the amount of unmet need is understandably higher in earlier years. In the right plot of IP80 using 80% of IP100, the number of people needing dedicated affordable housing is particularly high and does not decrease for many years. The number of people needing rapid rehousing also does not appear to approach zero in the short term.

This analysis encourages us to formulate an alternative investment policy that allocates more resources to the pathways with the largest queues, while decreasing resources to those with lower queues that reach zero more quickly. This tests the sensitivity of IP100 to small changes in the resource allocations. We attempt to stay around the $2.5 billion cost associated with the investment policy in Table 3 while finding an allocation that decreases the overall numbers in the queue in early years.

Consider increasing the investment in rapid rehousing, dedicated affordable housing, and permanent supportive housing by 10% of the values in Table 3. These are the pathways with the longest queues, but also have fairly high costs per unit. To reduce the overall costs down to $2.5 billion over five years, we reduce the number of planned units for rapid resolution (RR-Long and RR-Short), youth housing, and permanent supportive housing for seniors to 80% of those of in IP100 in Table 3. Call this investment policy IP1080.

**Fig 8:**
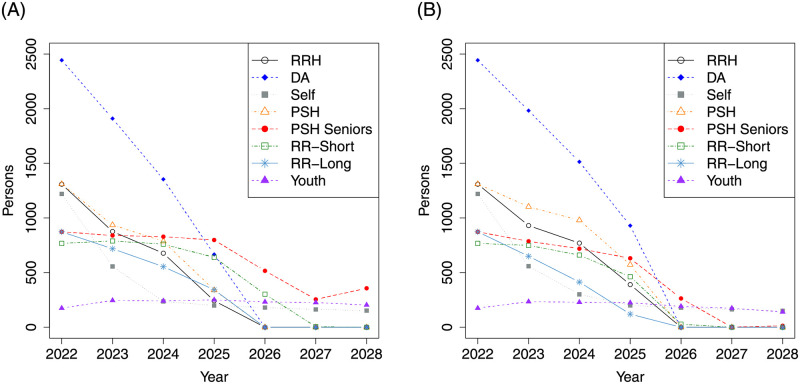
Left: Unmet need using investment policy IP1080 which increased investment to some pathways by 110% and decreased others to 80% of IP100. Right: Unmet need using investment policy IP0590 which increased investment to some pathways by 105% and decreased others to 90% of IP100.

The left plot of Fig 8 shows the results of investment policy IP1080. The rate of decrease in unmet need for DA is faster than in IP100, but the decrease in resources allocated to other areas means that many of the pathways do not go to zero. We can also consider increasing investment in rapid rehousing, dedicated affordable housing, and permanent supportive housing by only 5% while decreasing investment in planned units for rapid resolution (RR-Long and RR-Short), youth housing, and permanent supportive housing for seniors to 90% of the original levels in IP100. The result of this policy (IP0590) is shown in the right plot of Fig 8, which does a slightly better job of bringing PSH-Senior and RR-Short down to zero earlier.

To summarize the policies tested, Table 4 lists the properties of each policy, as well as the cost to build the desired inventory and total number of people with an unmet need each year. This total unmet need sums over the values for each pathway in the prior figures. The first three policies have similar costs but different allocations relative to IP100, with IP1080 and IP0590 investing more in rapid rehousing, dedicated affordable housing, and permanent supportive housing which is more expensive but has the most current need, while decreasing investment in the other pathways. Focusing on these current populations with large numbers of unsheltered does decrease the total amount of people with unmet need in the early years (in IP1080 and IP0590 compared to IP100), but we end up with more people left in the system in later years due to underinvestment in other pathways. A constant effort to reallocate resources according to current needs would lead to better results, rather than using the results from the most recent year to project forward multiple years.

**Table 4:** Summary of policy costs and total unmet need.

Policy Name	Scale Factors (Up/down)	Cost (billions)	Total Unmet Need (Persons)					
			2023	2024	2025	2026	2027	2028
IP100	1.00	$2.46	7,125	5,780	3,725	354	292	231
IP1080	1.10/0.80	$2.51	6,870	5,440	3,478	1,231	650	713
IP0590	1.05/0.90	$2.49	6,994	5,587	3,531	662	339	303
IP90	0.90	$2.23	10,584	7,693	6,480	2,386	561	317
IP80	0.80	$2.00	11,467	13,409	15,103	15,575	10,681	10,424

The investment plans IP90 and IP80 are less expensive due to the overall smaller investment levels across all pathways. In particular, investing only 80% of the baseline case is not enough to bring the unmet need down to manageable levels. Given the high queueing instability in the system, it will take a large influx of resources to bring down the current queue to stable levels. Once the system is somewhat stable, it may be beneficial to analyze the effects of investing in short-term versus long-term housing. But given the current state of Alameda County, almost all resources require large amounts of immediate investment to be even to begin to approach a feasible solution. However, all the policies in this paper assume the rate of arrivals remain approximately the same as recent levels over the five years (subject to non-homogeneous Poisson fluctuations). If prevention was able to significantly reduce the number of people emerging as homeless, then perhaps the required investment long-term could be drastically reduced.

## 6 Conclusion

We construct two simulation models for the flow of people through Alameda County’s homelessness response system. The models incorporate data input estimates and proposed investment policies to predict the number of people in the system over time needing housing and shelter. The first model treats all pathways through the system as homogeneous to estimate the total amount of housing and shelter needed over time. The second model differentiates between the various needs and pathways through the system and allows for testing different investment allocation policies. Overall, it is clear that a substantial increase in new housing inventory is needed both to address the current number of unsheltered people, and to manage future inflow of people to the system. An increase in shelter would help in the near term to mitigate some of the suffering faced by the unsheltered homeless population, but without new housing resources, shelter alone does not result in a long-term solution. In particular, investment in longer-term solutions such as dedicated affordable housing and permanent supportive housing is needed.

Estimates of future inflow to the system remain highly uncertain. If there is a rapid rise in homelessness in coming years due to impacts from the COVID-19 pandemic, economic instabilities or otherwise, current resources will be hugely inadequate. But should this rate of inflow decrease, future work would be able to easily recalibrate the simulation model to determine new levels of housing needed in the system. The current study focused on attempting to determine how to reach functional zero in five years, but did not take into account the realities of what would be needed in order to generate the type of funding and resources called for in the model.

The aggregate model can be easily applied to other settings where there are similar permanent and temporary resources, for example, the process of foster care (temporary housing) and adoption (permanent housing) for children. Of course, homeless populations in regions other than Alameda County can be considered by changing the data inputs for the aggregate model. In fact, other regions may not face the same levels of instability as Alameda County and thus may be able to achieve their objectives with more ease and less cost.

As for the detailed model, different regions will have varying pathways available depending on unique needs. In this case, the model should be modified to add and remove pathways as needed and ensure the logic representing the flow of people through the system is accurate. Finally, the simulation model can be easily adapted to work with any investment policy depending on the resources available at different time periods. The model should be updated each year based on new knowledge of the number of people in each stage of the system in order to provide updated predictions. Future work will test the effect of converting shelter into permanent housing in the long term. We anticipate many potential innovations in this space.
